# Dual‐line Genome‐scale CRISPR Screening Enables Robust Target Gene Discovery

**DOI:** 10.1002/advs.77130

**Published:** 2026-08-11

**Authors:** Ziniu Li, Jiao Qiao, Qiuyuan Zhang, Peipei Liu, Chenjun Zheng, Qi Zhou, Mengqi Ji, Zhihui Zhu, Xiaoxue Li, Hongyu Zhang

**Affiliations:** ^1^ National Key Laboratory For Germplasm Innovation and Utilization for Fruit and Vegetable Horticultural Crops Hubei Hongshan Laboratory Institute of Urban and Horticultural Entomology College of Plant Science and Technology Huazhong Agricultural University Wuhan People's Republic of China; ^2^ Hubei Insect Resources Utilization and Sustainable Pest Management Key Laboratory College of Plant Science and Technology Huazhong Agricultural University Wuhan People's Republic of China

**Keywords:** *Bactrocera dorsalis*, cold tolerance, CRISPR screening, dual‐line screening, functional genomics

## Abstract

Pooled CRISPR screening enables genome‐scale functional gene discovery by linking genetic perturbations to cellular phenotypes. However, its application in insects remains limited, as systematic differences often obscure the universal functional signals and compromise the reliable identification of genetic regulators. We found that Cas9 genomic integration was associated with distinct transcriptional states, editing activity, and screening outcomes. To address this limitation, we developed a dual‐line CRISPR screening framework that prioritizes genes with concordant effects across independently derived Cas9‐expressing insect cell lines. In *Bactrocera dorsalis*, dual‐line consensus screening increased in vivo validation of candidate genes from 60% in single‐line screens to 90%. Under cold stress, this framework identified candidate regulators of cold tolerance, including a previously uncharacterized gene whose perturbation altered embryonic survival at low temperature. These results show that dual‐line screening can filter cell line‐specific artifacts and improve functional genomic screening for reliable genetic target discovery in insects.

## Graphical abstract

A species‐optimized CRISPR platform integrates efficient *piggyBac* delivery, genome‐scale sgRNA libraries, and parallel screening in two independently engineered *Bactrocera dorsalis* Cas9 cell lines. Cross‐line consensus analysis filters line‐specific effects, enriches candidates with reproducible in vivo phenotypes, and reveals conserved, species‐specific, and cold‐responsive regulators for reliable genetic target discovery.

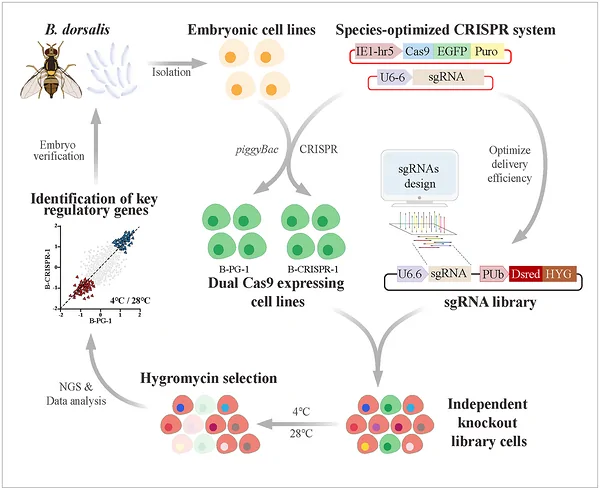

## 1 Introduction

Insect pests cause substantial losses in global crop production and pose serious threats to food security due to their wide distribution and population dynamics in response to climate change [1, 2]. Chemical insecticides remain the primary means of control but are increasingly constrained by resistance evolution and environmental concerns [3, 4, 5]. These limitations are driving the development of genetic control strategies, including RNA interference and CRISPR‐based approaches, which enable targeted disruption of genes essential for insect survival and offer more sustainable alternatives [6, 7]. Successful implementation of these strategies depends on molecular targets that produce strong phenotypic effects and are either conserved within target pest populations or sufficiently species‐selective to reduce non‐target risks. However, functional annotation is still incomplete for many insect genomes [8, 9]. In addition, many uncharacterized genes probably encode species‐specific essential functions that are not captured in current annotations. These limitations underscore the urgent need for high‐throughput functional screening to enable genome‐scale gene discovery.

Pooled CRISPR screening has emerged as a powerful strategy for identifying essential regulators of cellular growth, differentiation, and survival at genome scale [10, 11, 12]. By simultaneously introducing thousands of single‐guide RNAs (sgRNAs), pooled knockout screens enable systematic validation of gene functions at a population scale. Compared with RNAi screening, this approach provides higher specificity and reproducibility as well as reduced false‐positive rates [13, 14, 15], thereby enhancing the accuracy of functional gene discovery. For example, it has been used to identify Visgun as a receptor for the prototypical *Photorhabdus luminescens* Tc toxin [16], demonstrating its potential in rapid and accurate identification of molecular targets. Stable exogenous sequence integration in insect CRISPR screens is typically achieved through phiC31 integrase‐mediated site‐specific recombination or *piggyBac* transposon‐based integration [17, 18]. However, phiC31 integrase‐based CRISPR screening is limited by the requirement for pre‑engineered attP landing sites and by the impact of endogenous pseudo‑attP sites, which can reduce insertion‐site predictability and complicate quantitative interpretation in pooled screens [19, 20]. In contrast, *piggyBac* is more broadly applicable but mediates semi‐random genomic integration of exogenous sequences, which introduces positional bias that may distort library representation and also makes quantitative interpretation of the pooled screens complicated [21, 22]. In many insect pests, high‐throughput screening is further constrained by limited availability of standardized cell lines and efficient delivery systems. These challenges underscore the field‐wide need for adaptable and robust screening frameworks suitable for diverse insects.

Genome‐scale CRISPR screening in mammalian cancer cells has demonstrated consistent dependency scores for pan‐essential genes across independent datasets, whereas context‐specific gene dependencies are less reproducible across different cell lines and experimental conditions [23, 24]. This variability reflects multiple sources of systematic noise. Some experimental studies have shown that sgRNAs vary in activity, with low‐efficiency guides reducing gene depletion signals and leading to false‐negative identification of essential genes [25, 26]. At the genomic level, copy number amplification induces gene‐independent effects that confound gene dependency scores despite the use of computational correction strategies [27, 28]. Moreover, in vivo CRISPR screening is often subjected to engraftment bottlenecks and microenvironmental constraints [29, 30], resulting in gene dependencies different from those identified in vitro. These factors limit the robustness and cross‐system transferability of CRISPR screening.

We find that independently engineered Cas9‐expressing lines exhibited distinct transcriptional states, editing activities and screening outcomes. To address this limitation, we establish a dual‐line CRISPR screening framework that combines optimized *piggyBac* delivery with parallel screening in two independent Cas9‐expressing cell lines. This design enhances the robustness of functional target identification compared with single‐line screening. In *Bactrocera dorsalis*, a key target of FAO/IAEA‐coordinated area‐wide integrated pest management programs [31, 32], this framework enables systematic assessment of both conserved and species‐specific gene functions, and facilitates the identification of potential targets for pest control.

## 2 Results

### 2.1 A Robust CRISPR/Cas9 Platform in *B. dorsalis* Embryonic Cells

Embryonic cell lines were established from *B. dorsalis* embryos and maintained under standard insect cell culture conditions. Following long‐term passaging, two independent embryonic cell lines were derived and designated as Bd‐Egg‐A and Bd‐Egg‐B (Figure 1A). Although both lines were established under the same culture conditions, they showed distinguishable cellular morphologies. Bd‐Egg‐B was predominantly composed of round cells, whereas Bd‐Egg‐A contained a higher proportion of spindle‐shaped cells (Figure 1A). Growth‐curve analysis showed no obvious difference between the two cell lines, and both entered the exponential growth phase on day 3 after seeding (Figure S1A). These observations suggest that the two lines differed mainly in cellular composition rather than overall proliferative capacity. Because embryonic primary cultures contain heterogeneous cell populations, the morphological differences between Bd‐Egg‐A and Bd‐Egg‐B likely reflect stochastic retention and expansion of different cell subpopulations during long‐term in vitro passaging rather than inconsistent culture conditions. Cytogenetic analysis at passage 30 confirmed the chromosomal stability (Figure 1B; S1B), and mycoplasma assays verified the culture purity (Figure S1C). The species identity was confirmed by mitochondrial cytochrome c oxidase subunit I (COX1) sequencing, which showed >99% identity to the reference *B. dorsalis* sequences (Figure S1D; Table S1). To verify the developmental origin, we assessed the expression of embryonic stage‐specific markers, which were identified by integrating our embryonic transcriptome with publicly available developmental datasets. Both Bd‐Egg‐A and Bd‐Egg‐B showed the expression of LOC105224692, a marker specifically transcribed in 6‐24‐h embryos (Figure S2A–C). These results establish Bd‐Egg‐A and Bd‐Egg‐B as stable embryonic cell lines suitable for downstream functional studies.

**FIGURE 1:**
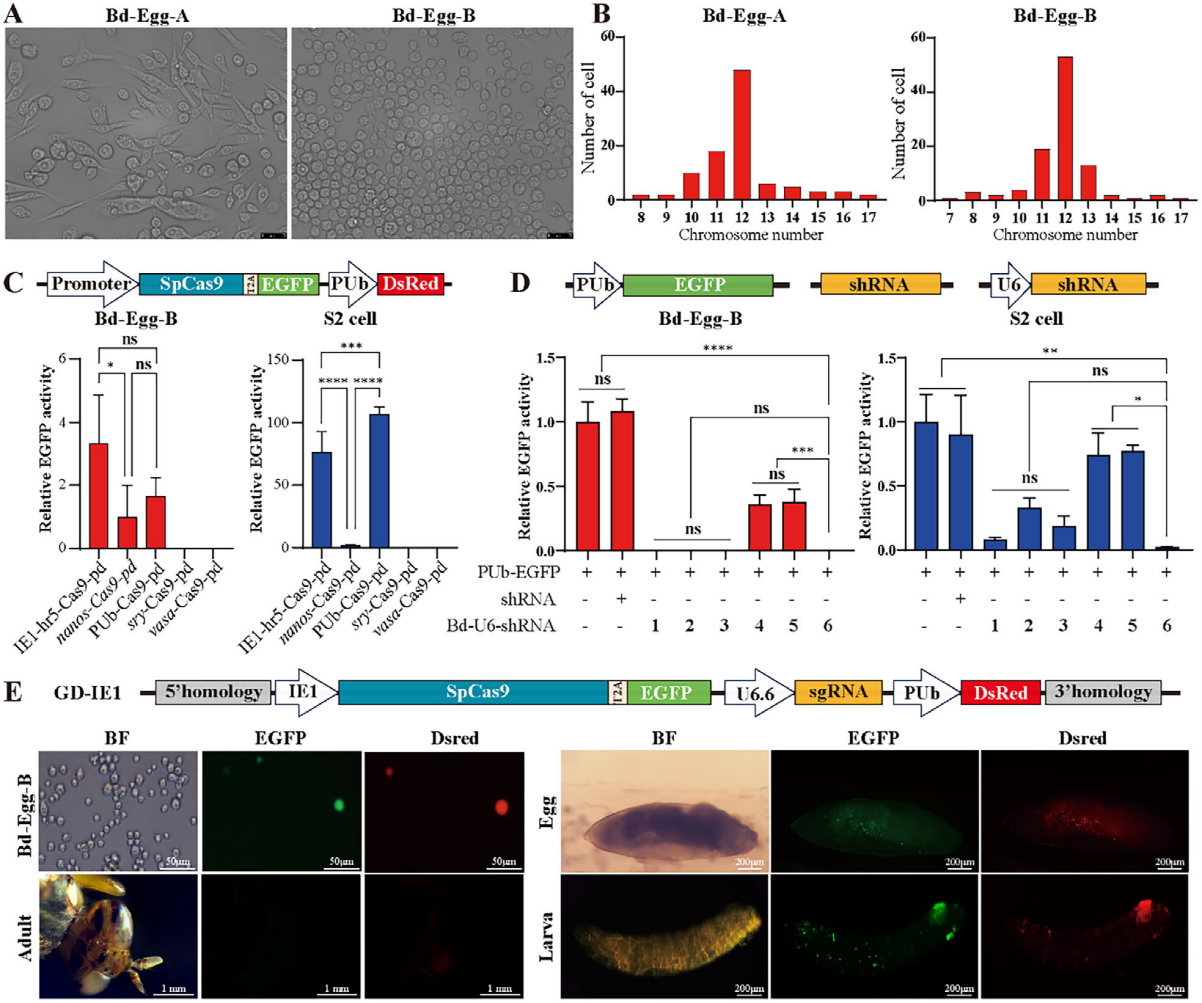
A CRISPR/Cas9 platform in *B. dorsalis* embryonic cell lines. A) Establishment of stable *B. dorsalis* embryonic cell lines. Bd‐Egg‐A and Bd‐Egg‐B cell lines were established after continuous culture of primary cells derived from 6–12 h embryos for more than six months. B) Karyotype analysis showing that the majority of cells contain six chromosome pairs. C) Cas9 expression driven by different promoters in Bd‐Egg‐B and S2 cells. Data are shown as the mean ± SD (n = 3). D) Activity of endogenous *B. dorsalis* U6 promoters was assessed by EGFP knockdown in Bd‐Egg‐B and S2 cells. “+” or “–” indicate the presence or absence of the indicated plasmid. Numbers indicate shRNA plasmids driven by the corresponding U6 promoters. Data are shown as the mean ± SD (n = 3). E) Functional validation of the CRISPR/Cas9 system using a homology‐directed repair (HDR) reporter targeting the *white* locus in cells and embryos. For all panels, statistical significance was determined by one‐way ANOVA followed by Tukey's multiple comparison test, *p < 0.05, **p < 0.01, ***p < 0.001, and ****p < 0.0001; ns, not significant.

To enable efficient genome editing in *B. dorsalis*, we next evaluated regulatory elements for Cas9 and sgRNA expression. Because Bd‐Egg‐B showed a more stable and relatively homogeneous morphology, which facilitated fluorescence observation and image‐based quantification, it was selected as the working cell line for these tool‐validation assays. Among five candidate Pol II promoters, the IE1‐hr5 and PUb promoters drove robust expression of EGFP, whereas endogenous promoters (*nanos*, *vasa*, and *sry*) showed minimal activity (Figure 1C; S3). In parallel, six endogenous U6 promoters from *B. dorsalis* were assessed using an EGFP‐shRNA knockdown assay as a functional readout of U6‐driven small RNA expression (Figure S4). Four constructs (Bd‐U6.1, Bd‐U6.2, Bd‐U6.3, and Bd‐U6.6) consistently mediated efficient EGFP knockdown in both Bd‐Egg‐B and S2 cells (Figure 1D; S5). Bd‐U6.6 was selected for subsequent shRNA and sgRNA expression constructs because it showed robust knockdown activity and had the shortest promoter fragment among the effective candidates. Finally, the plasmid‐based CRISPR/Cas9 system was evaluated using a reporter‐editing construct targeting the *white* gene (LOC105224216). The GD‐IE1 plasmid combined the IE1‐hr5 promoter for Cas9 expression with the Bd‐U6.6 promoter for sgRNA expression and contained *B. dorsalis white*‐derived left and right homology arms for HDR‐mediated targeting (Figure 1E). The donor constructs used in this study contained identical *B. dorsalis white*‐derived homology arms for HDR‐mediated targeting, and detailed sequences of all identically named elements are provided in Table S4. After either transfection into Bd‐Egg‐B cells or microinjection into early embryos, punctate EGFP and DsRed fluorescence was observed in some cells and embryos, with persistent signals through subsequent larval development. Although stable HDR‐mediated targeted integration was not established in embryos under the tested conditions, G0 adults exhibited obvious mosaic eye pigmentation (Figure 1E). Sequencing of the *white* locus confirmed the presence of InDels at the targeted sites, indicating that the GD‐IE1 construct could efficiently mediate mutagenesis (Figure S6). Collectively, these results establish a functional plasmid‐based CRISPR/Cas9 framework in *B. dorsalis* that supports targeted gene editing in both in vitro and in vivo contexts, while stable HDR‐mediated knock‐in will require further optimization.

### 2.2 Independent Cas9‐Expressing Lines Exhibit Distinct Cellular States

Despite the establishment of a functional CRISPR/Cas9 framework, it remained unclear whether independently engineered Cas9‐expressing cell lines would exhibit comparable cellular states and genome‐editing activities. To address this, CRISPR‐HDR and *piggyBac* strategies were used to generate Cas9‐expressing *B. dorsalis* cells (Figure 2A). Initial analyses revealed that cell lines differed in their ability to maintain Cas9 expression after antibiotic withdrawal. The proportion of Cas9‐positive cells declined rapidly in Bd‐Egg‐A cells, whereas Bd‐Egg‐B cells maintained a high fraction of Cas9‐positive cells and were used for subsequent analyses (Figure 2B). Within the Bd‐Egg‐B background, the Cas9‐expressing populations generated by CRISPR‐HDR and *piggyBac* showed different long‐term expression stability during continuous culture. Bd‐Egg‐B‐CRISPR cells consistently retained a high proportion of Cas9‐positive cells despite the presence of residual plasmids, but Bd‐Egg‐B‐PG cells showed a gradual loss of Cas9‐positive cells over time (Figure S7A,B).

**FIGURE 2:**
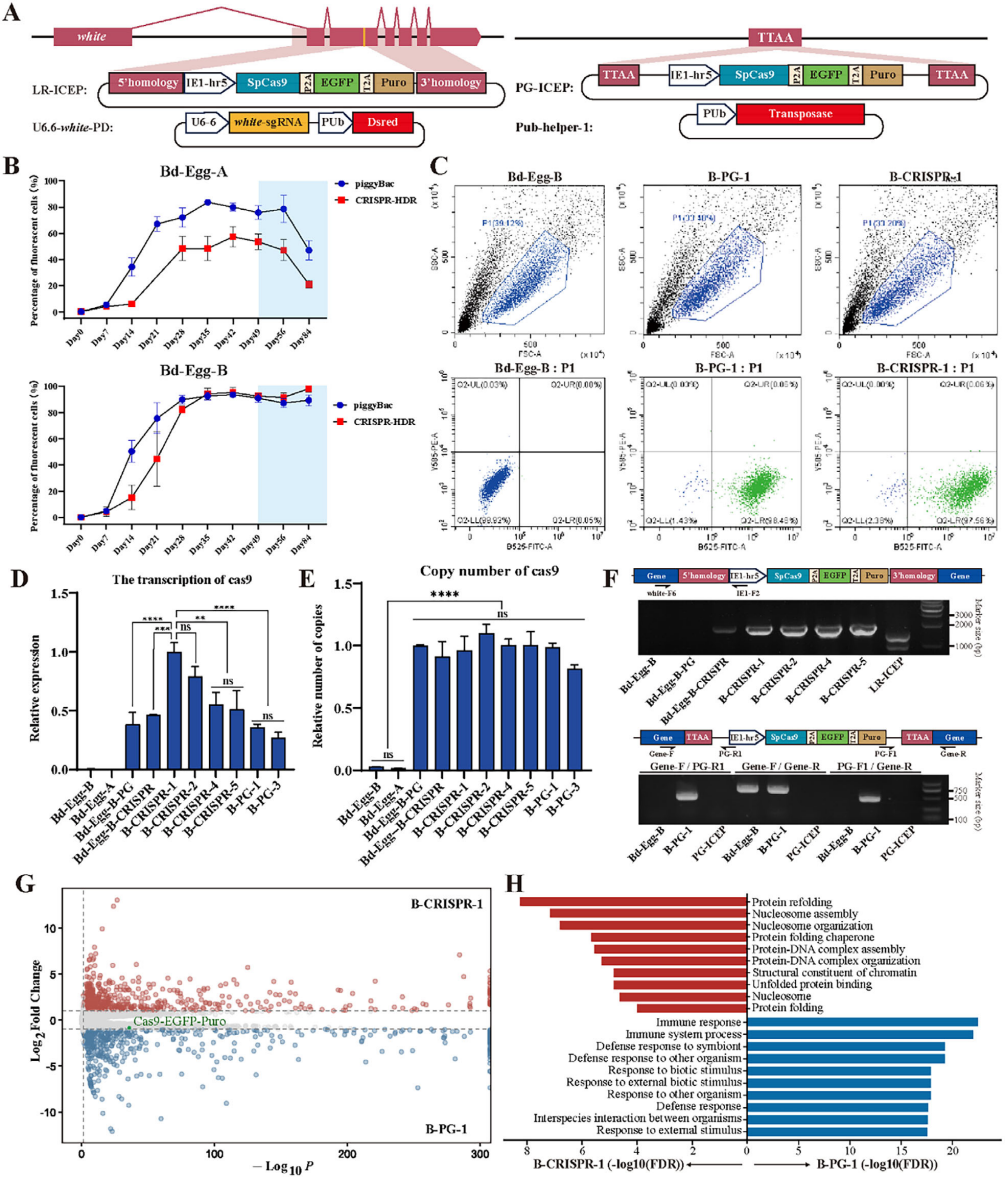
Independent Cas9 cell lines with distinct cellular states. A) Schematic overview of Cas9 integration strategy using CRISPR‐mediated HDR or *piggyBac* transposition. B) Growth dynamics of Cas9‐positive cellular populations. The white background indicates cultures sustained under persistent puromycin selection, whereas the light blue coloring indicates growth after antibiotic cessation. C) Flow cytometric analysis of purified Cas9‐positive cell lines. The P1 gate denotes the live cell population. D, E) Quantification of relative Cas9 expression by RT‐qPCR (D) and genomic Cas9 copy number across cell lines by qPCR (E). Data are shown as mean ± SEM (n = 3), **p < 0.01, ***p < 0.001, ****p < 0.0001; ns, not significant. Statistical significance was assessed by one‐way ANOVA with Tukey's multiple comparison test. F) PCR confirmation of Cas9 genomic integration sites. Arrows indicate primer positions. G) Differential gene expression analysis comparing B‐CRISPR‐1 and B‐PG‐1 cell lines. Volcano plot showing log_2_ fold change vs ‐log_10_ adjusted *p* value. Genes with |log_2_ fold change| > 1 and adjusted *p* < 0.05 were considered as significant. Red points indicate genes upregulated in B‐CRISPR‐1 cells, whereas blue points indicate genes upregulated in B‐PG‐1 cells. The Cas9‐EGFP‐Puro expression cassette showed no significant difference in transcription between the two cell lines. H) Functional enrichment analysis of differentially expressed genes between B‐CRISPR‐1 and B‐PG‐1 cell lines using KEGG pathway enrichment and GO analysis. The top 10 significantly enriched pathways are shown.

To enhance the stability of the CRISPR screen, we further purified Cas9‐expressing cells via limiting dilution. All selected clones exhibited uniformly high Cas9 positivity and showed no detectable plasmid contamination (Figure 2C; S7B,C). B‐CRISPR‐1 displayed the highest level of Cas9 transcription, whereas B‐PG‐1 and B‐PG‐3 showed the lowest Cas9 transcription (Figure 2D). To determine whether Cas9 transcript abundance reflected protein abundance, we further examined Cas9 protein levels by western blotting. Cas9 protein abundance was generally consistent with the mRNA levels, with B‐PG‐1 showing lower Cas9 protein abundance than B‐CRISPR‐1 (Figure S7D). However, EGFP disruption under identical conditions yielded a higher editing efficiency in B‐PG‐1 cells than in B‐CRISPR‐1 cells (Figure S7E). These results indicate that the editing efficiency of Cas9‐expressing lines cannot be explained solely by Cas9 transcript or total protein abundance. Copy number analysis indicated no significant difference in the genomic abundance of the Cas9 cassette among different lines (Figure 2E), suggesting that copy number is not the principal driver of differential Cas9 expression. As expected, the Cas9 cassette in B‐CRISPR cells was integrated into the third exon of the *white* gene (Figure 2F). Conversely, *piggyBac*‐mediated insertions were random, and the Cas9 cassette was inserted within the first intron covering LOC105224266 and LOC105224267 as confirmed through combined genome sequencing and PCR validation in B‐PG cells (Figure 2F; S7F). The residual wild‐type fragments at the target locus in B‐PG‐1 cells reflected the inherent mechanism of *piggyBac* insertion, which occurred on a single chromosome, leaving the homologous allele unmodified in clonally propagated cells.

To assess the potential effects of constitutive Cas9 expression in *B. dorsalis* cells, we examined both cellular growth characteristics and transcriptional states in parental Bd‐Egg‐B cells and the two Cas9‐expressing lines. Growth‐curve analysis showed comparable proliferation among Bd‐Egg‐B, B‐CRISPR‐1, and B‐PG‐1 cells under the same culture conditions (Figure S8A), and Trypan blue exclusion assays showed no significant difference in cell viability among the three cell lines (Figure S8B). Transcriptome profiling further showed that Cas9‐expressing cells were clearly separated from parental cells by principal component analysis (PCA), indicating distinct transcriptional states despite the absence of detectable proliferation or viability defects (Figure S8C). The differentially expressed genes between Cas9‐expressing and parental cells were notably enriched in pathways associated with immune signaling and metabolic regulation (Figure S8D,E). We further compared two independent Cas9‐expressing cell lines harboring Cas9 cassettes at distinct genomic loci. Genes upregulated in B‐PG‐1 cells were enriched in immune and defense‐related pathways, whereas those upregulated in B‐CRISPR‐1 cells were enriched in nucleosome assembly, chromatin organization, and protein‐folding pathways (Figure 2G,H). Together, these results suggest that engineered Cas9‐expressing lines can maintain normal proliferation and viability while exhibiting distinct transcriptional states that may contribute to cell line‐specific differences in genome‐editing activity.

### 2.3 Optimized *piggyBac* Delivery Enables High‐Coverage sgRNA Library Integration

Screening libraries can be typically categorized into knockout and interference libraries. To develop an appropriate screen system in *B. dorsalis* cells, we created four candidate vectors targeting EGFP with the *piggyBac* or CRISPR‐HDR insertion methods (Figure 3A). Four plasmid combinations were tested in B‐CRISPR‐1 and B‐PG‐1 cells for one month, including (a) PG‐EGFP‐sg‐PDH and PUb‐helper‐1, (b) PG‐EGFP‐sh‐PDH and PUb‐helper‐1, (c) LR‐EGFP‐sg‐PDH and U6.6‐*white*‐PD, and (d) LR‐EGFP‐sh‐PDH and U6.6‐*white*‐PD (Figure 2A; 3A). Flow cytometry of equivalent‐volume samples demonstrated that the first two groups sustained significantly greater cell densities than the latter two groups (Figure 3B; S9A), demonstrating that *piggyBac*‐mediated insertion is considerably more efficient than CRISPR HDR. Moreover, the PG‐EGFP‐sg‐PDH and LR‐EGFP‐sg‐PDH plasmids abolished over 97% of the EGFP fluorescence, greatly outperforming the PG‐EGFP‐sh‐PDH and LR‐EGFP‐sh‐PDH plasmids. These results indicated that sgRNA‐containing knockout vectors can achieve more efficient and stable target gene disruption. In B‐CRISPR‐1 cells, the combinations of LR‐EGFP‐sg‐PDH and LR‐EGFP‐sh‐PDH resulted in little enrichment of viable cells (Figure S9A), probably because the Cas9 cassette had already occupied the targeted insertion site, thereby obstructing the insertion of the library vector. Collectively, these results identified PG‐EGFP‐sg‐PDH as the most effective library vector for subsequent experiments.

**FIGURE 3:**
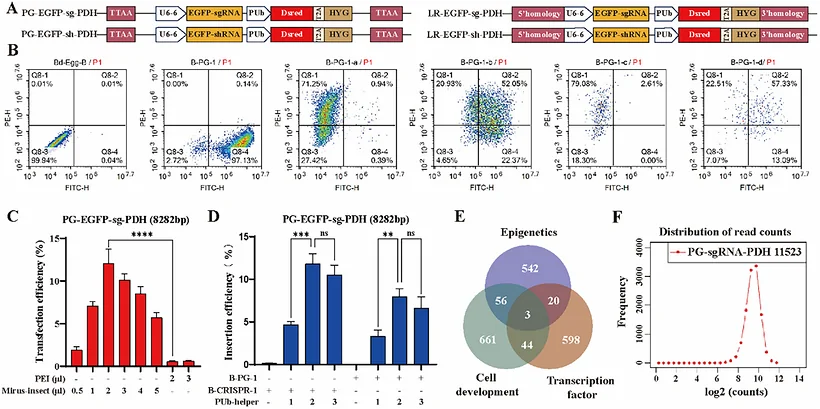
Optimized *piggyBac* delivery supports sgRNA library integration for pooled screening. A) Schematic of four candidate plasmid constructs for library development. B) Flow cytometric analysis of B‐PG‐1 cells transfected with four candidate vector combinations using *piggyBac* or HDR strategies. Equal sample volumes were analyzed across groups. C) Transfection efficiency of PEI‐MW40000 and TransIT‐Insect reagents. Data are shown as mean ± SEM (n = 6); ****p < 0.0001. D) Relative activity of three *piggyBac* transposases in Cas9‐expressing cells. Data are shown as mean ± SEM (n = 4), **p < 0.01, ***p < 0.001; ns, not significant. Statistical significance was determined by one‐way ANOVA with Tukey's multiple comparison test. E) Gene categories represented in the sgRNA library, including epigenetic regulators, cell developmental genes, and transcription factor networks. F) Next‐generation sequencing of the PG‐sgRNA‐PDH library showing uniform sgRNA distribution and comprehensive coverage.

To enhance the vector insertion efficiency, we first re‐evaluated the effectiveness of different transfection chemicals. In our earlier experiments, the commercially available reagent PEI‐MW40000 was routinely used for transfection, which exhibited a higher efficiency than several tested alternative reagents (Figure S9B), but its performance remained insufficient for large‐scale pooled CRISPR screening. Therefore, we performed a systematic screening to identify more effective transfection chemicals. TransIT‐Insect increased the plasmid delivery by approximately 13 folds compared with PEI‐MW40000 (Figure 3C). At a ratio of 1500 ng plasmid to 1.5 µl reagent, the transfection efficiency of TransIT‐Insect in 24‐well plates reached up to 23% (Figure S9C). Subsequently, we evaluated the efficacy of three *piggyBac* transposases to promote genomic insertion in *B. dorsalis* Cas9 cells. During *piggyBac* transposition, the target element was inserted into the host genome, whereas the residual circular plasmids remained as extrachromosomal molecules. The insertion efficiency was quantified by measuring plasmid abundance before and after integration using locus‐specific primers (Figure S9D; Table S1). The results indicated that PUb‐helper‐2 and PUb‐helper‐3 could enhance the insertion efficiency by over twofold compared with PUb‐helper‐1 (Figure 3D).

Based on these optimizations, we developed and synthesized an extensive sgRNA library comprising 1,925 genes linked to EGFP, epigenetic control, developmental processes, and transcription factor networks (Figure 3E). The library contained 11,523 sgRNAs, with an average of six sgRNAs per gene (Table S2). Next‐generation sequencing of the synthesized PG‐sgRNA‐PDH plasmid library verified a uniform plasmid distribution without any plasmid loss (Figure 3F). These results indicate that the library is suitable for subsequent CRISPR‐based functional target screening.

### 2.4 Dual‐line CRISPR Screening Enables Predictive Accuracy

Parallel pooled CRISPR screens were conducted using the PG‐sgRNA‐PDH library in two independently derived Cas9‐expressing *B. dorsalis* lines (B‐PG‐1 and B‐CRISPR‐1) to assess the consistency of screening outcomes across lines (Figure 4A). sgRNA abundance was quantified by sequencing after 30 or 60 days of continuous hygromycin selection, and gene‐level depletion was analyzed using the MAGeCK RRA algorithm. Biological replicates within each cell line showed high consistency (Figure S10), whereas screening outputs were markedly different between the two lines (Figure 4C; S11). Consistent with this observation, we identified 281 and 269 candidate essential genes in B‐PG‐1 and B‐CRISPR‐1 cells, respectively (FDR < 0.05), and only 85 genes were shared between the two screens (Figure 4B). PCA further revealed strong within‐line reproducibility but clear separation between cell lines (Figure S12). These results indicated that the observed variability is primarily driven by intrinsic differences between cell lines rather than by technical noise.

**FIGURE 4:**
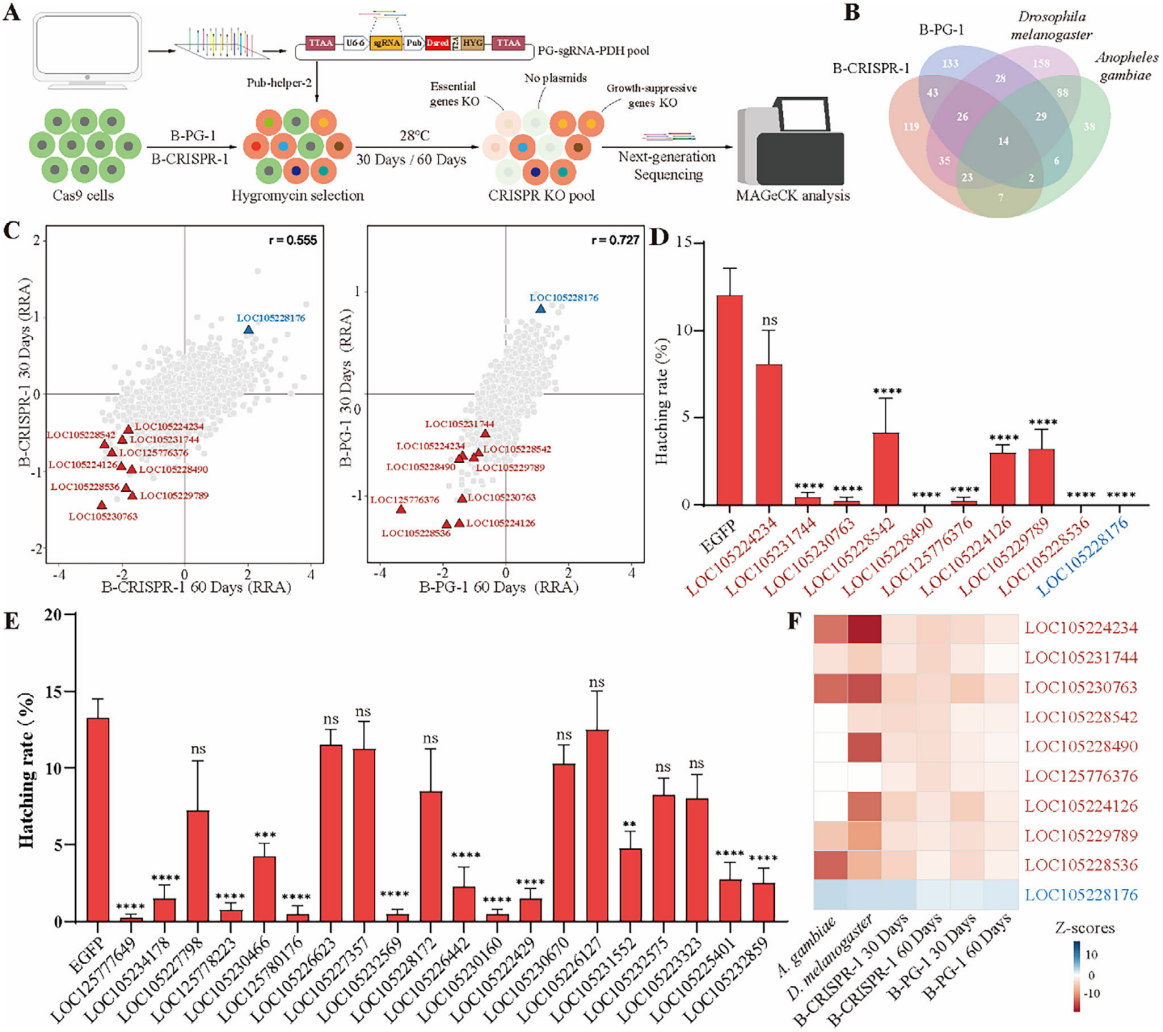
Dual‐line CRISPR screening enriches for developmental regulators with reproducible in vivo phenotypes. A) Schematic of the pooled CRISPR screening workflow. B) Comparative analysis of essential genes identified via CRISPR screens in *B. dorsalis*, *D. melanogaster*, and *A. gambiae*. C) Identification of dual‐line consensus essential and growth‐suppressive genes. The four comparisons comprised B‐PG‐1 at 30 and 60 days and B‐CRISPR‐1 at 30 and 60 days. Red triangles indicate genes meeting the negative‐selection empirical FDR < 0.05 threshold in all four comparisons, whereas blue triangles indicate genes meeting the positive‐selection empirical FDR < 0.05 threshold in all four comparisons. Values represent RRA scores. r denotes the Pearson correlation coefficient. D, E) Embryonic hatching rates following low‐dose injection of gene‐specific shRNA expression plasmids (30 ng/µl) into *B. dorsalis* embryos. An EGFP‐targeting plasmid was used as a negative control. (D) Genes identified as shared hits in dual‐line consensus screening. (E) Genes identified as single‐line specific hits. F) Conservation analysis of dual‐line screen hits across dipteran species. Values represent MLE Z‐scores. Data are shown as mean ± SEM (n = 4). Statistical significance was evaluated by one‐way ANOVA followed by Dunnett's multiple comparison test, **p < 0.01, ***p < 0.001, ****p < 0.0001; ns, not significant.

Considering this systematic discrepancy, we established a stringent dual‐line consensus criterion requiring consistent results in both B‐PG‐1 and B‐CRISPR‐1 cells at 30 and 60 days. Consensus essential genes were required to be significantly depleted in all four comparisons, whereas consensus growth‐suppressive genes were required to be significantly enriched (FDR < 0.05). Application of these criteria identified nine consensus essential genes and one consensus growth‐suppressive gene (Figure 4C). The essential genes included LOC125776376 (*Adf‐1*), LOC105228536 (*DIMT1*), LOC105228490 (*APC11*), LOC105224126 (*VhaAC39‐1*), LOC105230763 (*RpL40*), LOC105224234 (*Usp7*), LOC105229789 (*Arp2*), LOC105228542 (*gpp*), and LOC105231744 (*TRM11*), and the growth‐suppressive gene was LOC105228176 (*Smurf*). To evaluate the predictive performance of these candidates, we performed in vivo embryonic interference assays. Knockdown of nine of the 10 consensus genes significantly reduced hatchability (Figure 4D). RT‐qPCR analysis showed significant transcript reduction for eight of the 10 consensus genes, whereas LOC105230763 and LOC105229789 did not show statistically significant knockdown under the current assay conditions (Figure S13A). Both genes nevertheless showed reduced hatchability in the embryo shRNA assay. For comparison, ten additional candidates were selected from the 60‐day negative‐selection results of each cell line. These genes met the essential‐gene criterion in the corresponding line but did not satisfy the full dual‐line consensus criterion (Table S6). Knockdown of 12 of these 20 genes affected the embryonic development (Figure 4E). Among these 20 candidates, RT‐qPCR analysis showed significant knockdown for 15 genes, whereas LOC105234178, LOC125778223, LOC105226442, LOC105222429, and LOC105226127 did not show statistically significant transcript reduction (Figure S13B). Among these five genes, four showed significant hatchability phenotypes, while one showed no detectable embryo phenotype under the current assay conditions. In silico off‐target analysis further showed that none of the 30 consensus‐gene or single‐line‐specific shRNAs had predicted non‐target transcripts with perfect full‐length matches (Table S5). These results indicate that dual‐line consensus screening enriches for candidates with reproducible in *vivo* phenotypes compared with matched single‐line specific candidates, even when shRNA knockdown efficiency is taken into account.

### 2.5 Conservation and Lineage Specificity of Essential Genes

To evaluate the evolutionary conservation of the screened candidate essential genes, we performed a comparative analysis of them with recently published CRISPR screens from closely related Dipteran species, including *Drosophila melanogaster* [17] and *Anopheles gambiae* [31]. Ensembl orthology mapping revealed that 1,925 genes screened in *B. dorsalis* corresponded to 1,299 *D. melanogaster* and 1,234 *A. gambiae* homologs, among which 401 and 207 had been previously characterized as essential genes, respectively (Figure 4B; Table S3). Although the overlap of essential genes across the three species was limited (66 genes), functional enrichment analysis revealed strong pathway‐level convergence. The essential genes were predominantly localized to the nucleus and cytoplasm and enriched in pathways central to cell growth, including transcriptional regulation, ubiquitination, Polycomb‐associated processes, endocytosis, and canonical signalling cascades such as FOXO, mTOR, and Wnt (Figures S14–S16). Notably, similar pathway enrichments were also observed in genome‐scale CRISPR screens in *B. mori* embryonic cells [32], suggesting that these regulatory networks play conserved roles in insect development and metabolism.

We next assessed the cross‐species conservation of ten essential genes identified by dual‐line screening. MAGeCK maximum likelihood estimation (MLE) revealed a high consistency in z‐score distribution for these genes across *B. dorsalis*, *D. melanogaster*, and *A. gambiae* (Figure 4F), indicating their high degrees of functional conservation among insect lineages. These findings further demonstrated the robustness of our integrated CRISPR screening framework. In addition, the genes at LOC125776376 locus had no detectable homolog in other species, and those at the LOC105228490 locus were also lack of identifiable homologs in *A. gambiae* (Figure 4F; Table S3). These results demonstrated the capacity of the framework to detect species‐specific genes besides functionally conserved orthologs.

### 2.6 Context‐dependent Genetic Regulators Associated With Cold Tolerance

To identify context‐dependent genetic regulators of cold tolerance, we performed CRISPR screening under cold stress conditions (Figure 5A). Following 30 days of library selection at 28°C, the cell populations were maintained at 4°C for an additional 30 days. This prolonged treatment was used as a stringent selection condition to reveal cumulative differences in cell survival among gene perturbations under sustained cold stress. Consistent with our previous results, screening profiles were highly reproducible within each cell line but differed substantially between the two lines (Figure 5B; S17). Dual‐line consensus screening identified 15 shared high‐confidence hits (FDR < 0.05), comprising eight essential and seven growth‐suppressive genes under cold stress (Figure 5B,D). For comparison, the union‐based candidate set included genes reaching FDR < 0.05 in at least one Cas9‐expressing line, while genes showing opposite directions of selection between lines were excluded. This relaxed criterion expanded the candidate space to 188 depleted and 210 enriched genes (Figure 5D). The union‐based candidates were enriched for nuclear functions and processes related to transcriptional regulation and ATP metabolism. Importantly, perturbation of genes in the Toll and Imd signaling pathways reduced cellular cold tolerance, whereas perturbation of genes involved in metal ion binding and ubiquitin‐mediated proteolysis enhanced cell survival under cold conditions (Figure 5C).

**FIGURE 5:**
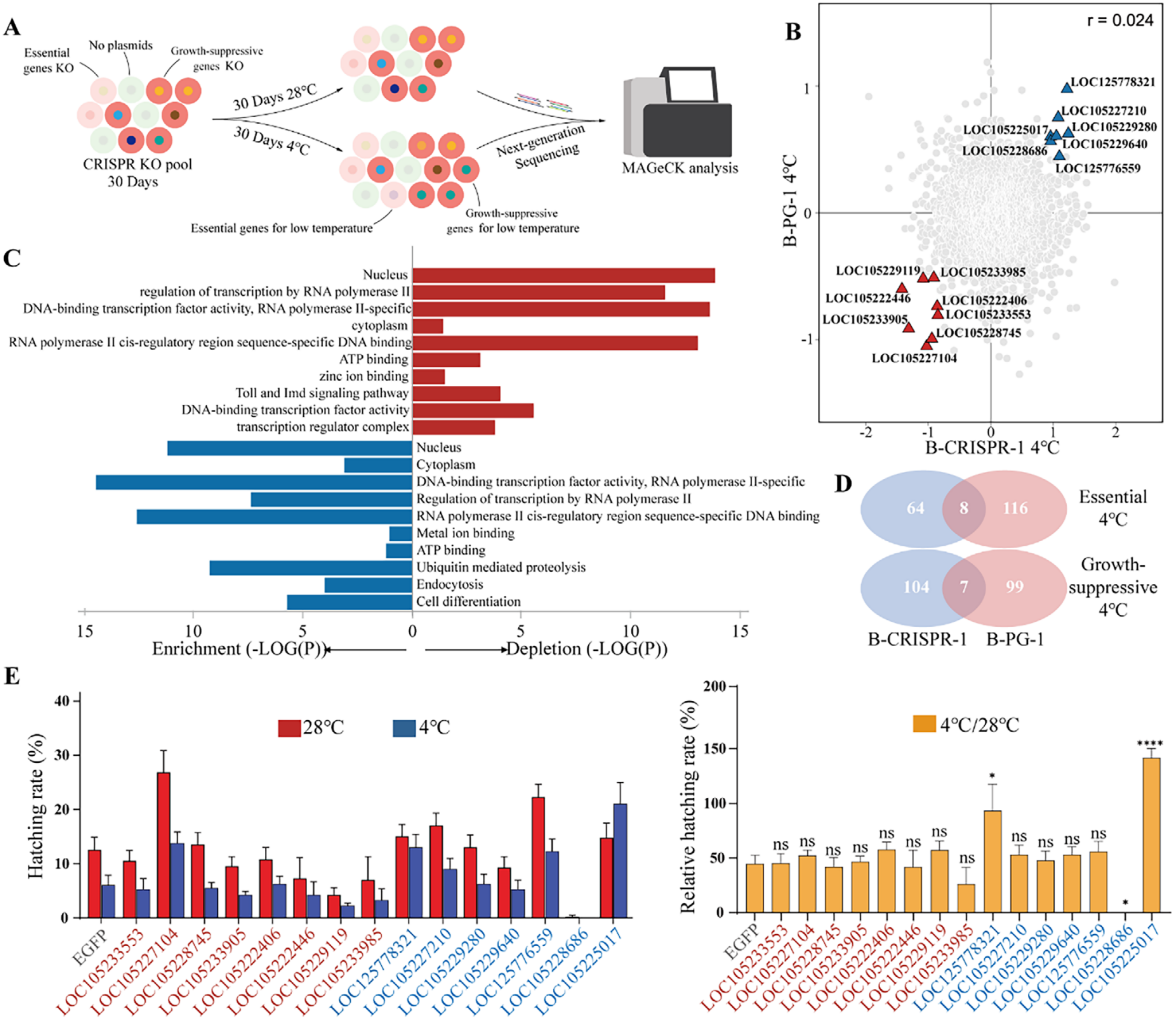
Dual‐line CRISPR screening to identify cold‐tolerance regulators and validate their functions in embryos. A) Schematic of pooled CRISPR screening under cold stress. The PG‐sgRNA‐PDH library was transduced into B‐PG‐1 or B‐CRISPR‐1 cells. Cells were maintained at 28°C for 30 days followed by 4°C for 30 days, generating B‐PG‐1 and B‐CRISPR‐1 populations under cold conditions. B) Identification of cold‐regulatory genes in B‐PG‐1 and B‐CRISPR‐1 cells. Red triangles indicate genes consistently classified as essential for cold adaptation across both datasets, whereas blue triangles indicate genes identified as cold‐growth suppressors. RRA scores from MAGeCK analysis are shown. r denotes the Pearson correlation coefficient. C) Functional annotation of cold‐regulatory genes in *B. dorsalis* using KEGG and GO enrichment analysis. The top 10 significantly enriched pathways are shown. D) Cold‐regulatory genes identified through pooled CRISPR screening. E) Hatching rates of *B. dorsalis* embryos following injection of gene‐specific shRNA expression plasmids targeting cold‐regulatory genes. Red bars indicate hatching rates at 28°C, blue bars indicate hatching rates following 4°C treatment, and yellow bars represent the 4°C/28°C hatching rate ratio. Data are shown as mean ± SEM (n = 4). Statistical significance was evaluated by one‐way ANOVA followed by Dunnett's multiple comparison test, *p < 0.05, ****p < 0.0001; ns, not significant.

Gene‐specific shRNA expression plasmids were microinjected into *B. dorsalis* embryos. Among the 15 cold‐associated candidates, RT‐qPCR analysis showed significant transcript reduction for 11 genes, whereas LOC105233553, LOC105222446, LOC105229119, and LOC125776559 did not show statistically significant knockdown under the current assay conditions (Figure S13C). Knockdown of LOC105225017 (*Highwire*) and LOC125778321 significantly increased hatchability under cold conditions, consistent with the cell‐based screening result, indicating that both genes act as negative regulators of cold tolerance (Figure 5E). LOC125778321 has not yet been functionally characterized in *B. dorsalis*. Interference with LOC105228686 led to embryonic lethality, corroborating its identification as a growth‐essential gene in the B‐CRISPR‐1 60‐day dataset (Figure 5E; Table S3). In contrast, silencing of the remaining candidates had no detectable effect on the hatching rate at low temperature. For the 15 cold‐associated candidates, potential shRNA off‐target effects were also assessed. Fourteen of the 15 shRNA constructs had no predicted non‐target transcripts with perfect full‐length matches. LOC125776559 was the only exception, with 17 predicted non‐target transcripts showing full‐length identity to the shRNA target sequence (Table S5). Together, these results link cell‐based cold‐stress screens to embryonic phenotypes and highlight candidate regulators relevant to temperature‐sensitive pest control.

## 3 Discussion

Genome‐scale CRISPR screening can systematically link genotypes to phenotypes and enable prioritization of functional targets beyond homology‐based inference [10, 17]. However, this approach has remained largely confined to a few model species due to limited cell line availability, poor delivery efficiency, and confounding insertion‐site effects [16, 31, 33]. Here, we established an adaptable pooled CRISPR screening workflow in *B. dorsalis* and found that independently engineered Cas9‐expressing lines showed distinct cellular states, editing efficiencies, and screening outcomes. These findings identify engineered cell‐line background as a potential source of line‐specific bias in pooled CRISPR screening. To address this, we developed a dual‐line screening strategy that leverages cross‐line concordance to mitigate the impact of line‐specific bias and enable consensus‐based identification of robust functional targets in *B. dorsalis*.

Variation among independently engineered clones may arise from multiple features of cell‐line construction. One potential source is the integration strategy. B‐PG‐1 and B‐CRISPR‐1 were generated through *piggyBac*‐mediated random integration and HDR‐mediated targeted integration, respectively. The *piggyBac* system preferentially integrates at TTAA tetranucleotides, allowing the cassette to randomly occupy different genomic and chromatin environments that can affect transgene expression and stability [34, 35]. Although HDR‐mediated integration improves positional control, CRISPR‐directed knock‐in can still produce unwanted integration events and heterogeneous repair outcomes [36]. Therefore, integration‐associated effects may contribute to the differences between the two lines. Notably, B‐PG‐1 showed lower Cas9 transcript and protein abundance than B‐CRISPR‐1 but higher EGFP disruption efficiency. This discrepancy indicates that editing output is not determined by Cas9 abundance alone. Integrated reporter studies have shown that genomic location can strongly influence Cas9‐induced mutation efficiency even when defined Cas9‐gRNA systems are used [37]. Local chromatin environment may explain this locus dependence, because nucleosome occupancy can restrict Cas9 binding and cleavage, whereas chromatin context can shape editing efficiency and repair outcomes after Cas9‐induced double‐strand breaks [38, 39]. In addition to integration‐site effects, the inserted cassette may further shape clone‐specific cellular states. In mammalian systems, guide‐free Cas9 expression has been associated with genomic damage and transcriptomic alterations, which suggests that stable Cas9 expression may affect the basal state of engineered cells [40, 41]. Consistent with this possibility, Cas9‐expressing cells in our study were transcriptionally distinct from parental cells and showed enrichment of immune and metabolic pathways. During subsequent pooled screening, sgRNA‐guided cleavage may further amplify these pre‐existing differences by activating DNA‐damage responses and selecting cells that better tolerate editing‐associated stress [42, 43]. GFP and selectable‐marker systems have also been reported to alter host expression profiles or the distribution of linked transgene expression [44], suggesting that cassette composition may add to integration‐associated variation.

Drug adaptation and clonal divergence may further diversify independently generated Cas9 lines. Puromycin and hygromycin B inhibit protein synthesis, and prolonged selection with these antibiotics can favor cells that better tolerate translational stress through altered transporter activity, protein‐homeostasis regulation, and stress‐response programs beyond the resistance conferred by the marker gene alone [45, 46, 47]. Clonal isolation can fix and reveal pre‐existing heterogeneity within a parental cell population. Consequently, independently derived clones retain distinct transcriptional or phenotypic states [48]. This phenomenon was particularly evident among the four independently isolated B‐CRISPR clones, which showed marked differences in Cas9 expression despite being generated using the same HDR‐based strategy. Together, these effects may reinforce clone‐specific cellular states and introduce background‐dependent variation into single‐line CRISPR screens.

A critical challenge for pooled CRISPR screening is achieving sufficient library representation and stable genetic perturbation [15], as low transfection efficiency has been shown to limit coverage and reduce reproducibility in insect systems [13, 32, 49]. We established an adaptable pooled CRISPR screening workflow in *B. dorsalis* by optimizing plasmid delivery and *piggyBac*‐mediated integration. Compared with the commonly used strategies, these improvements increased the plasmid uptake by approximately 13‐fold and transposon insertion by more than twofold. As the workflow relies on plasmid transfection and transposase‐mediated insertion, it circumvents the requirements for viral packaging or pre‐engineered genomic landing sites [13, 19, 33], which are often difficult to implement in non‐model insect systems. A streamlined and efficient CRISPR screening platform is better positioned for adoption in insect and non‐model systems. However, an important consideration for *piggyBac*‐based pooled delivery is that sgRNA integration multiplicity is not intrinsically restricted to one cassette per cell. In a genome‐wide *piggyBac* CRISPR screen in *B. mori*, single‐cell sequencing showed that more than 95% of cells carried a single sgRNA insertion under optimized conditions [32]. In contrast, another *piggyBac* CRISPR‐library delivery system recovered clones carrying multiple distinct integrated gRNA constructs, and the distribution of integration multiplicity varied with the amount of *piggyBac* library DNA used for transfection [50]. Because sgRNA integration multiplicity was not assessed at the single‐cell level in the present study, variable numbers of integrated sgRNA cassettes and the co‐integration of distinct sgRNAs could not be excluded.

False‐positive and background‐dependent signals remain persistent challenges in pooled CRISPR screens [51, 52]. Variable sgRNA integration multiplicity can introduce additional bias by confounding the assignment of fitness effects to individual sgRNAs and generating combinatorial perturbation effects [53]. Together with the engineered‐line differences described above, variability in sgRNA integration may have contributed to the low concordance between the two Cas9‐expressing lines and the accumulation of line‐specific candidate signals. Consistency across cell lines can filter out dependencies driven by background or stochastic effects, thereby enriching for genes whose perturbation reflects intrinsic biological function and produces consistent phenotypes in vivo [54, 55]. This dual‐line strategy substantially improves candidate identification, with nine out of ten high‐confidence developmental regulators significantly reducing embryonic hatchability, representing an approximate 30% increase in predictive accuracy relative to matched single‐line‐specific candidates. In addition, large‐scale insect CRISPR screens often generate extensive candidate lists that complicate downstream in vivo validation and increase attrition during whole‐organism phenotypic testing [15, 17, 31, 32]. By prioritizing precision and interpretability over maximal recall, dual‐line screening provides an efficient route from in *vitro* fitness profiling to candidate targets with reproducible in *vivo* phenotypes, thereby enabling more predictive target discovery in pest insects.

Beyond demonstrating the robustness of the framework, the screens also exhibited both conserved and species‐specific genetic dependencies. Under a dual‐line consistency criterion, we consistently identified the core essential genes in *B. dorsalis*, whose orthologs retain conserved developmental functions in *D. melanogaster* and *A. gambiae* [17, 31, 56]. Even under relaxed thresholds, there was still robust pathway‐level conservation, with enrichment of ribosome biogenesis, RNA metabolism, and cell‐cycle regulation [57]. In addition, the framework was used to successfully detect lineage‐specific candidates such as *Adf‐1*, suggesting that the framework can prioritize candidates that may be missed by homology‐based approaches. This value is exemplified by the Y‐linked gene *typo‐gyf* in *B. dorsalis*, whose functions in male survival and spermatogenesis were validated by RNAi and CRISPR/Cas9 [58]. In contrast, conventional target discovery approaches heavily rely on gene homology to model organisms and the prioritization of evolutionarily conserved genes [59, 60], which may overlook the lineage‐specific vulnerabilities. Because our framework interrogates gene function empirically, it can identify candidate genes even when the genome annotations are incomplete or when the genes lack clear orthologs [61, 62]. Redeploying the screening pipeline under cold stress further revealed regulators associated with cellular cold tolerance, including ubiquitin‐proteasome‐related factors and a previously uncharacterized gene (LOC125778321) validated in embryos. The functional importance of this pathway in *B. dorsalis* is further supported by the UPS‐dependent regulation of the Lolal–*dpp* axis during reproduction [63]. Notably, most cold‐associated regulators identified in cell‐based screens had no detectable effects on embryonic hatchability in vivo. This pattern is consistent with prior studies showing that genetic dependencies revealed under environmental stress often reflect condition‐specific cellular fitness requirements rather than baseline developmental essentiality [64, 65].

These features make the framework suited for nucleic acid‐based pest control strategies, where effective targets must possess both functional impacts and species specificity [66]. Since the organism‐level validation in this study was limited to embryogenesis under controlled laboratory conditions, extending assays to additional life stages, tissues, and environmental contexts will be important to assess the ecological relevance [18, 32]. Future refinements may be focused on systematic rescue experiments or coupling defined genetic perturbations with multi‐omics profiling to strengthen the causal inference and refine the underlying regulatory networks [38, 67, 68]. The framework is also compatible with layered design workflows in which in silico tools are used to prioritize candidate sequences. Transcriptome‐informed off‐target prediction frameworks, such as dsRNAEngineer [69], can be used for guide library design, while pooled CRISPR screens can be employed for genome‐scale functional validation and ranking. Overall, this framework not only enables scalable functional genomics in *B. dorsalis* but also provides a framework that may be adapted for genome‐scale target discovery in other pest insects, which is significant for both fundamental research and environmentally responsive pest management.

## 4 Conclusion

Pooled CRISPR screening can be limited by line‐specific cellular states and perturbation variability, particularly in non‐model systems with immature genetic toolkits. Here, we show that Cas9 integration context was associated with altered transcriptional states and divergent screening outputs in *B. dorsalis* embryonic cells. By prioritizing cross‐line concordance, dual‐line pooled CRISPR screening enriched candidates with reproducible in *vivo* phenotypes and identified conserved as well as cold‐tolerance regulators. This strategy prioritizes reproducible genetic effects and enables robust target discovery for pest functional genomics.

## 5 Experimental Section

### 5.1 B. Dorsalis Rearing

*B. dorsalis* was maintained under controlled environmental conditions at 27°C and 70 ± 5% relative humidity, with a 12: 12 h light: dark photoperiod. Larvae were reared on a semi‐artificial diet composed of 500 g banana, 500 g cornmeal, 100 g sucrose, 100 g yeast, and 100 g paper, which were thoroughly mixed with 1 L of water to achieve a homogeneous substrate. Adults were fed with an artificial diet consisting of yeast and sucrose in a 1: 3 ratio. All rearing procedures were conducted at the Institute of Horticulture and Urban Entomology, Huazhong Agricultural University (Wuhan, China).

### 5.2 Cell Isolation and Culture

A continuous cell line was established from 6–12 h *B. dorsalis* eggs. The eggs were sequentially surface‐sterilized by immersion in 10% sodium hypochlorite solution for 5 min, followed by 75% ethanol for another 5 min, and then rinsed three times with sterile distilled water. Sterilized eggs were transferred into sterile tubes containing 1 mL of TNM‐FH (Procell Life Sciences & Technology Co., Ltd, Wuhan, China) medium supplemented with 10% fetal bovine serum (10270‐106, Thermo Fisher Scientific). Using a sterile rubber pestle, the eggs were gently ground to release cells. The homogenate was filtered through a 100 µm cell strainer to remove debris, and the filtrate was transferred into a 25 cm^2^ culture flask containing 5 mL of the same culture medium. Cultures were maintained at 28°C in a humidified incubator without CO_2_ supplementation. Primary cells isolated directly from eggs were cultured with medium changes every 10 days until a stable, long‐term proliferating cell line was established. Subsequently, the medium was replaced every three days to maintain cell growth. Mycoplasma contamination was routinely examined using the GMyc‐PCR Mycoplasma Detection Kit (Yeasen, Shanghai, China) according to the manufacturer's instructions.

### 5.3 Cell Proliferation and Viability Assays

Cells were seeded at the same initial density and cultured under identical conditions. Cell density was determined daily for 7 days to generate growth curves. For the viability assay, Bd‐Egg‐B, B‐PG‐1, and B‐CRISPR‐1 cells were seeded at approximately 50% confluence under identical culture conditions. Cells were collected and mixed with an equal volume of 0.4% Trypan blue solution. After incubation for 4 min at room temperature, unstained viable cells and blue‐stained nonviable cells were counted using a hemocytometer. Cell viability was calculated as viable cells/(viable cells + nonviable cells) × 100%. Three independent biological replicates were performed for each cell line.

### 5.4 Cell Transfection

Cells were seeded and cultured until reaching approximately 80% confluence. Prior to transfection, the culture medium was replaced with serum‐free TNM‐FH medium to optimize transfection efficiency. Plasmid DNA was complexed with one of the following commercially available transfection reagents according to the respective manufacturers’ protocols: Lipofectamine 2000 (Thermo Fisher Scientific, 11668019), Lipo6000 (Beyotime, C0526), Lipo8000 (Beyotime, C0533), LipoInsect (Beyotime, C0551), PEI‐MW40000 (YEASEN, 40816ES03), or TransIT‐Insect (Mirus, MIR6100). For each reagent, plasmid DNA and transfection reagent were diluted separately in serum‐free medium, combined, and incubated at room temperature for 20 min to allow complex formation. The DNA‐reagent complexes were then added to the cells, which were incubated for 10 h before adding an equal volume of TNM‐FH medium supplemented with 20% fetal bovine serum (FBS). Cells were further cultured for 48 to 72 h before downstream analyses. Negative controls included cells treated with transfection reagent alone or with empty vector to confirm transfection specificity. The transfection efficiency was assessed qualitatively by fluorescence microscopy and quantitatively by flow cytometry measuring reporter gene expression.

### 5.5 Cell Identification

To confirm the identity of the established cell line, genomic DNA was extracted from cultured cells and subjected to PCR amplification targeting the mitochondrial cytochrome c oxidase subunit I (COX1) gene. The primer sequences used were Bd‐COX1‐F (5’‐CGACAATGGTTATTCTCAACAAATCA‐3’) and Bd‐COX1‐R (5’‐TTAAATCCATTGCACTAATCTGCCA‐3’). PCR products were purified and subjected to Sanger sequencing. The resulting sequences were compared and validated by BLAST analysis against the NCBI database to confirm the origin of the cell line.

### 5.6 Chromosome Karyotype Analysis

Chromosome karyotyping was performed on cultured cells at metaphase to assess chromosomal number and morphology. *B. dorsalis* is a diploid species with six pairs of chromosomes (2n = 12). Cells were first treated with colchicine (0.2 µg/mL) for 3–4 h to accumulate metaphase cells. Subsequently, cells were harvested and subjected to hypotonic treatment with 0.5% potassium chloride (KCl) for 20 min to swell the cells and spread chromosomes. Cells were then fixed three times in freshly prepared Carnoy's fixative (methanol: acetic acid, 3:1), each fixation lasting 15 min. Fixed cells were dropped onto clean glass slides, air‐dried, and stained using Giemsa solution for 30 min to visualize chromosomes. Chromosome spreads were examined under a light microscope at 1000× magnification using oil immersion. For each cell line, 100 randomly selected analyzable metaphase spreads were photographed and counted to summarize the number distribution. Only metaphase spreads in which individual chromosomes could be reasonably distinguished were used for chromosome counting.

### 5.7 Plasmid Construction

Two plasmids of the *piggyBac* transposon system were kindly provided by Dr. Alfred M. Handler, including the transposon vector pB[PUb_DsRed.T3] and the transposase plasmid phspBac. To generate various expression constructs, DNA fragments were ligated to the pBac vector using the Seamless Cloning Kit (Beyotime, D7010S). Specifically, the original PUb‐DsRed cassette located between the XmaI and SacII restriction sites was replaced with different expression cassettes, including promoter‐SpCas9‐T2A‐EGFP, PUb‐EGFP, IE1‐hr5‐SpCas9‐P2A‐EGFP‐T2A‐Puro, and U6.6‐sgRNA‐PUb‐DsRed‐T2A‐HYG. For the transposase plasmid phspBac, the native hsp70 promoter was substituted with the PUb promoter to create the PUb‐helper‐1 construct. Additionally, two other transposase variants, PUb‐helper‐2 and PUb‐helper‐3, were constructed by cloning the transposase coding sequences from the hyperactive transposases hyPBase apis [70] and Super *piggyBac* Transposase expression vector (System Biosciences, PB210PA‐1), respectively, under the control of the PUb promoter. For CRISPR/Cas9‐mediated HDR, donor plasmids were constructed using the pTOPO‐TA vector (CV2201, Aidlab). The donor constructs contained the desired insert flanked by homology arms corresponding to the target locus: a 1469 bp 5’ homology arm and a 1500 bp 3’ homology arm, enabling precise genomic integration. The SpCas9, EGFP, puromycin resistance (Puro), hygromycin resistance (HYG), and sgRNA scaffold were sourced from the pLV3‐U6‐MCS‐sgRNA‐Cas9‐EGFP‐Puro and pCDH‐CMV‐MCS‐EF1a‐CopGFP‐T2A‐Hyg plasmids (MiaoLing Plasmid Platform). These fragments were assembled via fusion PCR with 20‐bp overlapping ends to ensure precise and efficient cloning. All cloning steps were performed following standard molecular biology protocols. Restriction enzyme digestions and ligations were carried out according to manufacturer instructions (NEB). PCR amplifications employed PrimeSTAR Max DNA Polymerase (TaKaRa, R047A) under the following cycling conditions: 35 cycles of 98°C for 10 s, 55°C for 15 s, 72°C for 10 s/kb. Constructs were verified by Sanger sequencing (Qingke, China). Gene‐specific shRNA expression plasmids used for embryo validation were constructed in the pTOPO‐TA backbone by replacing the EGFP‐targeting sequence in the U6.6‐shRNA cassette with a 20‐nt sequence complementary to the transcript of each candidate gene. The shRNA cassette was driven by the *B. dorsalis* U6.6 promoter and was expected to mediate RNAi‐dependent knockdown of the target transcript. The target sequences are provided in Table S5. Detailed information for the plasmids and key functional elements used in this study, including promoter sequences, sgRNA/shRNA sequences, selectable markers, transposase coding sequences, and left/right homology arm sequences, is provided in Table S4.

### 5.8 Nucleic Acid Extraction, Reverse Transcription, and qPCR

According to the supplier's instructions, genomic DNA was extracted from samples using the Animal tissue DNA kit (Simgen, 3101050), and total RNA was isolated using RNAiso Plus reagent (Takara, 9108). The quality and concentration of extracted DNA and RNA were assessed by spectrophotometric analysis and verified by 1% agarose gel electrophoresis to confirm integrity and absence of degradation. Purified DNA samples were stored at −20°C for long‐term preservation, while RNA samples were stored at −80°C until further use. Complementary DNA (cDNA) was synthesized from 1 µg of total RNA using the PrimeScript FAST RT reagent Kit with gDNA Eraser (Takara, RR092A). Quantitative real‐time PCR (qPCR) was performed on a CFX96 Real‐Time PCR Detection System (Bio‐Rad) using appropriate primers and SYBR Green mix. The RPL32 was used as an internal reference to normalize gene expression levels. Relative gene expression was calculated using the 2^−ΔΔCT^ method [71]. All reactions were conducted in technical triplicates with appropriate no‐template controls to ensure specificity and reproducibility. The information of relative primer sequences was listed in Table S1.

### 5.9 Identification and Evaluation of U6 Promoters

To identify endogenous U6 promoters in *B. dorsalis*, the *D. melanogaster* U6.1 (CR32867) small nuclear RNA (snRNA) sequence was used as a query to perform a BLAST search against the *B. dorsalis* reference genome (GCA_023373825.1) in the NCBI database. Complete snRNA sequences along with 600 bp upstream regions were extracted and aligned using Jalview software for multiple sequence alignment and visualization. This enabled the identification of conserved RNA polymerase III promoter motifs, specifically the proximal sequence element A (PSEA) and TATA box, characteristic of Pol III‐driven U6 promoters. Among the identified promoters, the U6.6 promoter (GenBank: FJ668652.1), previously utilized in Bddsx^++f^‐shRNA expression vectors [72], served as a reference. For functional evaluation of endogenous U6 promoters from *B. dorsalis*, six candidate U6 promoter sequences were PCR‐amplified from genomic DNA. Each promoter fragment was fused to a short hairpin RNA (shRNA) targeting EGFP via overlap‐extension PCR, generating U6‐shRNA cassettes. These cassettes were subsequently cloned seamlessly into the pTOPO‐TA vector to produce U6‐EGFP‐shRNA expression plasmids. As a negative control, an EGFP‐targeting shRNA fragment without any U6 promoter was directly cloned into pTOPO‐TA to generate an EGFP‐shRNA plasmid. 500 ng of each U6‐EGFP‐shRNA plasmid was mixed with 500 ng of the PUb‐EGFP reporter plasmid and transfected into cultured cells in 24‐well plates using 2 µL of PEI‐MW40000 transfection reagent. Control groups included co‐transfection of 500 ng EGFP‐shRNA plasmid with 500 ng PUb‐EGFP plasmid, as well as transfection with 500 ng PUb‐EGFP plasmid alone. EGFP fluorescence was monitored using fluorescence microscopy, and representative images were captured. The density of EGFP‐positive cells was quantified with ImageJ software to assess the relative transcriptional activity of each U6 promoter based on shRNA‐mediated knockdown efficiency.

### 5.10 Flow Cytometry Analysis

Insect cells were collected and transferred into 15 mL centrifuge tubes, followed by centrifugation at 100×g for 5 min at room temperature. The supernatant was carefully discarded, and the cell pellet was gently resuspended in phosphate‐buffered saline (PBS). This washing step was repeated twice to remove residual debris and culture medium. Subsequently, the cell suspension was filtered through a 70 µm nylon mesh to eliminate aggregates and ensure a single‐cell suspension suitable for flow cytometric analysis. Flow cytometry was performed using a CytoFLEX LX flow cytometer (Beckman Coulter) equipped with 488 and 561 nm lasers. Fluorescence signals corresponding to EGFP and DsRed expression were detected in the FITC channel (488 nm excitation, 525/40 nm emission) and PE channel (561 nm excitation, 585/42 nm emission), respectively. Forward scatter (FSC) and side scatter (SSC) parameters were used to gate viable cell populations and exclude debris. For each sample, a minimum of 10,000 events were acquired to ensure statistical robustness. The data acquisition and subsequent analysis were performed using CytExpert software.

### 5.11 RNA‐Seq and Data Analysis

Total RNA was extracted from samples and subsequently submitted to the Novogene for library preparation and sequencing as previously reported [73]. Briefly, RNA libraries were constructed following Illumina's standard protocols and sequenced on the Illumina NovaSeq X Plus platform to generate 150 bp paired‐end (PE) reads, ensuring high depth and coverage for comprehensive transcriptome profiling. Raw sequencing reads underwent stringent quality control, including adapter trimming and removal of low‐quality bases, to obtain clean reads. These clean reads were aligned to the reference genome of *B. dorsalis* (GenBank accession: GCA_023373825.1) using HISAT2 with default parameters, enabling accurate spliced alignment and efficient mapping. Gene‐level quantification was performed using featureCounts, which counts reads mapped to annotated exons, generating raw counts for downstream analysis. Differential gene expression analysis was conducted using the DESeq2 R package, which normalizes count data and models gene expression changes using a negative binomial distribution. Genes with |log2 FC| > 1 (FDR < 0.05) were considered significantly differentially expressed. Visualization of expression patterns was achieved through heatmaps generated by the pheatmap R package, while volcano plots illustrating the distribution of fold changes and statistical significance were produced using the EnhancedVolcano package. Functional enrichment analyses for GO and KEGG pathways were performed using DAVID. Enriched terms with *P* ≤ 0.05 were considered statistically significant and visualized using the ggplot2 R package.

### 5.12 Embryo Microinjection

Embryos of *B. dorsalis* were collected within 15 min post‐oviposition and dechorionated by immersion in 1.2% sodium hypochlorite for 1 min, followed by rinsing with 0.02% Triton X‐100 to remove the chorion. Dechorionated embryos were immobilized on glass slides using double‐sided tape for microinjection. Microinjections were performed using an air‐injection apparatus (Pneumatic PicoPump PV820, World Precision Instruments, USA) within 1 h post oviposition. Plasmid solutions were prepared at 300 ng/µL for the GD‐IE1 plasmid and 30 ng/µL for the gene‐specific shRNA expression plasmid. Injection volumes were calibrated to 1%–2% of embryo volume to minimize mechanical damage while ensuring effective nucleic acid delivery. Post‐injection, embryos were overlaid with Halocarbon oil 700 to prevent desiccation and incubated at 28°C in a humidified incubator to support normal embryogenesis.

### 5.13 Construction and Synthesis of the sgRNA Library

A sgRNA library targeting 1,925 genes involved in cellular processes, environmental information processing, transcriptional regulation, and epigenetic regulation was designed using Cas‐Designer. Candidate sgRNAs were designed against the *B. dorsalis* reference genome assembly ASM2337382v1 (GCF_023373825.1) and NCBI Annotation Release 103, with the SpCas9 NGG PAM setting. Candidate sequences were retained only when they mapped to a single exact genomic position and did not contain a continuous TTTT motif. Eligible sgRNAs were ranked using a composite CRISPR score. The ranking prioritized target sites located in earlier coding exons, greater transcript‐isoform coverage, an out‐of‐frame score of at least 66, and a GC content between 20% and 80%. For genes with multiple annotated transcripts, sgRNAs targeting all transcripts were assigned the highest priority, followed by those targeting at least 50% of the transcripts. Candidates were ranked first according to the composite CRISPR score and then according to the out‐of‐frame score. Candidate sgRNAs were initially designed using all annotated CDS regions of each gene as the target sequence. When fewer than six eligible sgRNAs were identified, the design region was expanded sequentially to annotated exon regions and then to the full gene region. Up to six top‐ranking sgRNAs were selected for each gene, resulting in a final library of 11,523 sgRNAs. Detailed sgRNA design information and definitions of the corresponding parameters are provided in Table S2. Oligonucleotide pools containing the designed sgRNA sequences were synthesized by Zhuhai Generulor Medical Technology company using array‐based single‐stranded DNA synthesis technology. These oligonucleotides were cloned into the PG‐EGFP‐SG‐PDH vector backbone to replace the original EGFP‐sgRNA sequence. sgRNA library was assessed by Illumina next‐generation sequencing, confirming the successful cloning of 11,523 sgRNAs with over 99.99% coverage.

### 5.14 CRISPR Screening and Data Analysis

B‐PG‐1 and B‐CRISPR‐1 cells were cultured in TNM‐FH medium at 28°C until reaching approximately 80% confluence. Cells were transfected in triplicate in 25 cm^2^ flasks using a mixture containing 10 µg of the sgRNA library, 10 µg of PUb‐helper‐2 plasmid, 20 µL of TransIT‐Insect reagent, and 1 mL of TNM‐FH medium. Seven days post‐transfection, hygromycin was added to the culture medium at 600 µg/mL to maintain continuous selection. Cells were harvested at 30 and 60 days after selection initiation. For the cold‐stress screen, B‐PG‐1 and B‐CRISPR‐1 cells carrying the sgRNA library were maintained at 28°C for 30 days under hygromycin selection and then transferred to 4°C for an additional 30 days. Parallel cell populations maintained at 28°C for the same period served as controls. For each replicate, cells were collected to ensure > 1000‐fold coverage per sgRNA. Genomic DNA was extracted using a previously established protocol. Integrated sgRNA sequences were subsequently amplified by a two‐step PCR. The first PCR used primers U6‐JC‐F1 and U6‐JC‐R1 for 30 cycles to generate the initial amplicon. The second PCR employed primers U6‐JC‐F2 and U6‐JC‐R2 for 30 cycles to append Illumina sequencing adapters and sample‐specific barcodes. Final amplicons were sequenced on an Illumina MiSeq PE150 platform (Tsingke Biological Technology, China), targeting a minimum sequencing depth of 500 reads per sgRNA. Sequencing reads were demultiplexed and mapped to the reference sgRNA library. MAGeCK RRA was used for primary gene‐level identification of positive and negative selection, whereas MLE‐derived Z‐scores were used for the cross‐species comparison shown in Figure 4F. An expression‐informed empirical FDR procedure was adapted from recent genome‐wide CRISPR screens in *D. melanogaster* and *A. gambiae* [17, 31]. Genes with TPM < 1 in the corresponding cell‐line transcriptome were classified as lowly or non‐expressed and used as an empirical false‐positive set. Consensus hits were called only when the corresponding empirical FDR was < 0.05 in both B‐PG‐1 and B‐CRISPR‐1 cells at 30 and 60 days, with depletion defining essential genes and enrichment defining growth‐suppressive genes.

### 5.15 Embryo Cold‐Validation Assays

For each biological replicate, a single batch of embryos injected with a given gene‐specific shRNA expression plasmid was randomly divided into control and cold‐treatment groups. At 8 h post‐injection, embryos in the cold‐treatment group were transferred to 4°C for 10 h and then returned to 28°C for continued development. Control embryos were maintained continuously at 28°C. Embryo hatching was continuously monitored after the cold‐treated embryos were returned to 28°C. Four independent injection batches were analyzed for each construct. Relative cold hatchability was calculated by dividing the hatchability following 4°C treatment by that of the corresponding 28°C control group.

### 5.16 Evaluation of piggyBac Transposition Efficiency

To evaluate the transposition efficiency of the *piggyBac* system, cells were co‐transfected with a *piggyBac* transposon vector (PG‐EGFP‐sg‐PDH) and a transposase‐expressing plasmid at a 1: 1 molar ratio. As a negative control, cells were transfected with an equimolar mixture of the *piggyBac* transposon vector and an EGFP‐shRNA plasmid lacking transposase expression. One week post transfection, the genomic DNA was extracted using Animal tissue DNA kit (Simgen, 3101050) to obtain high‐quality nucleic acid suitable for qPCR analysis. As illustrated in Figure S9, the total amount of the plasmid vector was quantified by qPCR using the PG‐F1/PG‐R1 primer pair, while the quantity of plasmids that had undergone transposition was determined using the M13‐F/M13‐R primer pair. *piggyBac* transposition efficiency (%) was defined as (quantity of M13 amplicon/quantity of PG amplicon) × 100. Primer sequences are listed in Table S1.

### 5.17 In Silico Prediction of shRNA OFF‐Target Effects

Potential off‐target effects of shRNA target sequences were evaluated using the updated *B. dorsalis* transcriptome (ASM2337382v1). Each shRNA target sequence was searched against the transcriptome using seqkit (v2.13.0) software, allowing 0, 1, 2, or 3 mismatches across the full target sequence. Matches corresponding to the intended target gene were removed, and the remaining matches were counted as predicted non‐target hits. Predicted non‐target transcripts with a perfect full‐length match were considered high‐risk off‐target candidates, whereas non‐target transcripts with one or two mismatches were considered potential off‐target candidates. The off‐target prediction results are provided in Table S5.

## Author Contributions

H.Z., X.L., and Z.L. conceived and designed the study. Z.L., J.Q, Q.Z., P.L. and C.Z. contributed to experimental materials and sample collection. Z.L., Q.Z. and M.J. analyzed the data. Z.L. wrote the manuscript. H.Z., X.L., and Z.Z. supervised the work and revised the manuscript. All authors read and approved the final version.

## Conflicts of Interest

The authors declare no conflicts of interest.

## Supporting information




**Supporting File 1**: advs77130‐sup‐0001‐SuppMat.docx.

**Supporting File 2**: advs77130‐sup‐0002‐S1.xlsx.

**Supporting File 3**: advs77130‐sup‐0003‐S2.xlsx.

**Supporting File 4**: advs77130‐sup‐0004‐S3.xlsx.

**Supporting File 5**: advs77130‐sup‐0005‐S4.xlsx.

**Supporting File 6**: advs77130‐sup‐0006‐S5.xlsx.

**Supporting File 7**: advs77130‐sup‐0007‐S6.xlsx.

## Data Availability

The data that supports the findings of this study are available in the supplementary material of this article.
