# Supplementary material for: Dual‐line Genome‐scale CRISPR Screening Enables Robust Target Gene Discovery
Source: Adv Sci (Weinh). 2026 Aug 11:e77130. Online ahead of print. doi: 10.1002/advs.77130 (PMC13460280; doi:10.1002/advs.77130)
Supplement: Supplementary file 1 [file ADVS-9999-e77130-s001.docx]

**Figure S1.** Characterization of *B. dorsalis* embryonic cell lines. A) Growth curves of Bd-Egg-A and Bd-Egg-B after seeding. B) Karyotype analysis of Bd-Egg-A and Bd-Egg-B. Most analyzed metaphase spreads showed six pairs of chromosomes. The Y chromosome (red arrow) was observed in a subset of clear metaphase spreads, suggesting that the cultures were derived from mixed-sex embryos. Representative male (♂) and female (♀) karyotypes are indicated. C) Mycoplasma detection confirming that both Bd-Egg-A and Bd-Egg-B cultures were free of contamination. D) Alignment of COX1 sequences. Bd-Egg-A, Bd-Egg-B, *B. dorsalis* *embryos* and *B. dorsalis* adult sequences were compared with reference *B. dorsalis* and *D. melanogaster* sequences from NCBI. Conserved bases identical to the reference *B. dorsalis* sequence are shown in red, whereas mismatches are shown in yellow.

**Figure S2.** Identification and validation of stage-specific marker genes during *B. dorsalis* development. A) Stage-specific marker genes identified across the egg, larval, pupal, and adult stages of *B. dorsalis* using transcriptomic data. B) Validation of stage-specific marker expression. Among the candidate genes screened, only LOC105224692 was transcribed in both Bd-Egg-A and Bd-Egg-B cells. The other markers were also expressed at the corresponding developmental stages in *B. dorsalis* (1 h, 4 h, 8 h eggs; L1–L3 larvae; pupae; adult female [F]; adult male [M]). C) Developmental expression of LOC105224692. This gene was specifically expressed in 6–24 h embryos but absent at other stages.

**Figure S3.** EGFP fluorescence from Cas9 expression plasmids. A) EGFP distribution in Bd-Egg-B cells 72 h after transfection with five Cas9-expression plasmids, observed by fluorescence microscopy. B) EGFP distribution in S2 cells under the same conditions as A.

**Figure S4.** Sequence alignment of *B. dorsalis* U6 promoters. Multiple sequence alignment highlighting proximal sequence element A (PSEA), TATA box, and U6 snRNA transcription start sites (red boxes).

**Figure S5.** EGFP fluorescence after U6-shRNA-mediated knockdown. A) EGFP distribution in Bd-Egg-B cells co-transfected with the PUb-EGFP expression plasmid and a U6-shRNA knockdown construct, visualized by fluorescence microscopy. B) EGFP distribution in S2 cells under the same conditions as A.

**Figure S6.** Sanger sequencing of the G0 *white* gene. sgRNA target sites are indicated by black underlines, PAM sequences by red dashed lines, and Cas9 cleavage sites by black dashed lines. The primers white-F6 and white-R1 were used for amplification (Table S1, Supporting Information).

**Figure S7.** Assessment of Cas9 cell line integrity. A) Flow cytometric analysis of Cas9-expressing cells cultured for two months without antibiotic pressure, confirming long-term stability of Cas9-positive populations. B) Steady lines with EGFP and DsRed signals visible under a fluorescence microscope. Functional Cas9 cassette elements are retained when EGFP is present, while cells containing the U6.6-white-PD construct are marked with DsRed. C) Flow cytometric analysis of purified Cas9-positive cell lines. The P1 gate denotes the live cell population. D) Western blot analysis of Cas9 protein abundance in B-CRISPR-1 and B-PG-1 cells. E) Assessment of genome editing efficiency in Cas9-expressing cell lines. CRISPR/Cas9-mediated knockout efficiency was quantified by loss of EGFP signal in B-CRISPR-1 and B-PG-1 cells. F) Schematic illustration of the Cas9 cassette integration site in B-PG-1 cells.

**Figure S8.** Evaluation of differences in parental and Cas9-expressing *B. dorsalis* cell lines.

A) Growth curves of Bd-Egg-B, B-PG-1, and B-CRISPR-1 cells under identical culture conditions. B) Trypan blue exclusion assay showing comparable cell viability among Bd-Egg-B, B-PG-1, and B-CRISPR-1 cells. Data are presented as mean ± SEM; ns, not significant. Statistical significance was assessed using two-tailed Student’s *t*-test. C) Principal component analysis (PCA) of transcriptome datasets from wild-type and Cas9-expressing lines, illustrating global transcriptional variation. D) Functional enrichment analysis of differentially expressed genes between wild-type and Cas9-expressing cell lines. The gene set comprises genes with consistent differential expression patterns across both volcano plots in (E). The top 10 KEGG and GO terms are shown. E) Differential gene expression analysis of B-CRISPR-1 and B-PG-1 compared with the parental Bd-Egg-B cell line. Volcano plot showing log₂ fold change versus –log₁₀ adjusted *P* value. Genes with |log₂ fold change| > 1 and adjusted *P* < 0.05 were considered significant. Red points indicate genes upregulated in Bd-Egg-B cells, whereas blue points indicate genes upregulated in B-CRISPR-1 or B-PG-1 cells. The Cas9-EGFP-Puro expression cassette showed no significant difference in transcription between the two cell lines.

**Figure S9.** Construction and optimization of the CRISPR screening library. A) Flow cytometry analysis of B-CRISPR-1 cells transfected with four candidate vector combinations. Equal volumes were analyzed across all groups. B) Comparison of transfection efficiency across different reagents. Bd-Egg-B cells were transfected with 1,000 ng plasmid DNA per well in 24-well culture plates using Lipofectamine 2000 (Lipo2000), Lipo6000, Lipo8000, LipoInsect, or PEI-MW40000 (PEI). C) Optimization of the plasmid-to-reagent ratio for TransIT-Insect transfection in 24-well plates. Plasmids and reagents were individually diluted before mixing, and the final transfection mixture was adjusted to a total amount of 100 μl per well. D) Schematic illustration and PCR-based validation of piggyBac transposition efficiency. Transposition efficiency was calculated as the ratio of newly synthesized plasmids to total plasmids, with M13-F/M13-R primers quantifying newly formed plasmids and PG-F2/PG-R2 primers detecting the total input plasmid pool (Table S1, Supporting Information).

**Figure S10.** Reproducibility of CRISPR screen samples. Pearson correlation coefficients (r) were calculated among biological replicates within each treatment group. Each group consisted of three independent replicates, labeled as A, B, and C.

**Figure S11.** Identification of developmental regulatory genes in B-PG-1 and B-CRISPR-1 cells. Developmental regulatory genes were identified in B-PG-1 and B-CRISPR-1 cell lines. Red triangles indicate genes significantly depleted in both B-PG-1 and B-CRISPR-1 cells at 30 and 60 days, whereas blue triangles indicate genes significantly enriched under the same conditions (empirical FDR < 0.05). V Values represent MAGeCK RRA scores obtained from three biological replicates per comparison. r indicates the Pearson correlation coefficient between datasets.

**Figure S12.** Principal component analysis (PCA) of CRISPR screen samples. PCA was performed to evaluate the global clustering patterns of CRISPR screen samples from B-PG-1 and B-CRISPR-1 cell lines. Red circles represent B-CRISPR-1 cells, and blue triangles represent B-PG-1 cells. Sample labels indicate experimental treatments: 1M, hygromycin selection for 30 days; 2M, hygromycin selection for 60 days; 4 ℃, low-temperature treatment at 4 ℃. The percentage of variance explained by principal components 1 (PC1) and 2 (PC2) is indicated on the corresponding axes.

**Figure S13.** RT-qPCR analysis of shRNA-mediated knockdown efficiency in *B. dorsalis* embryos. A) Knockdown efficiency of the 10 dual-line consensus genes identified in both Cas9-expressing cell lines. B) Knockdown efficiency of the 20 single-line specific candidates selected from the B-PG-1 and B-CRISPR-1 screens. C) Knockdown efficiency of the 15 cold-associated candidate genes. Data are shown as mean ± SEM (n=3), **P* < 0.05, ***P* < 0.01, ****P* < 0.001, *****P* < 0.0001; ns, not significant. Statistical significance was assessed using two-tailed Student’s *t*-test.

**Figure S14.** KEGG and GO enrichment analysis of essential genes in *D. melanogaster*. The top 10 enriched terms are shown. CC, Cellular Component; MF, Molecular Function; BP, Biological Process.

**Figure S15.** KEGG and GO enrichment analysis of *B. dorsalis* homologs of 1,924 essential genes identified in *B. dorsalis*. The top 10 enriched terms are shown. CC, cellular component; MF, molecular function; BP, biological process.

**Figure S16.** KEGG and GO enrichment analysis of *A. gambiae* homologs of 1,924 essential genes identified in *B. dorsalis*. The top 10 enriched terms are shown. CC, cellular component; MF, molecular function; BP, biological process.

**Figure S17.** Reproducibility of CRISPR screen samples under 4 ℃. Pearson correlation coefficients (*r*) were calculated among biological replicates within each treatment group. Each group consisted of three independent replicates, labeled as A, B, and C.
